# Editorial: Personal competences in the academic and work environment: advancing towards psychological wellbeing

**DOI:** 10.3389/fpsyg.2025.1648176

**Published:** 2025-07-21

**Authors:** María del Mar Molero Jurado, María del Carmen Pérez-Fuentes, Carol D. Ryff, Pablo Molina Moreno

**Affiliations:** ^1^Department of Psychology, University of Almeria, Almería, Spain; ^2^Institute on Aging / Department of Psychology, University of Wisconsin-Madison, Madison, WI, United States

**Keywords:** psychological wellbeing, personal competencies, emotional intelligence, self-efficacy, academic and work environment, cultural engagement, mental health promotion

## 1 Introduction

Psychological wellbeing and individual development are core aspects of human development, involving ongoing processes of self-realization and life satisfaction. In this context, personal competencies—such as emotional intelligence, self-esteem, resilience, assertiveness, and autonomy—are recognized as essential resources for maintaining healthy psychological functioning. These skills support adaptability to change, regulate stress effectively, and navigate everyday challenges, thereby fostering a more fulfilling and meaningful life across personal, academic, and professional areas.

In addition to these competencies, cultural, artistic, and humanities-based experiences have shown transformative potential, enabling expression, self-reflection, and meaning-making processes that enhance psychological wellbeing. Wellbeing is thus understood not merely as the absence of psychological distress but as an active, dynamic journey of personal growth and fulfillment throughout the lifespan. The integration of personal competencies and enriching life experiences provides a promising foundation for the design of interventions, educational programs, and preventive strategies aimed at strengthening mental health and promoting personal growth.

This Research Topic provides an in-depth analysis of the ways in which personal competencies and cultural and artistic engagement contribute to psychological wellbeing from a positive perspective. It brings together empirical studies that, through an integrative lens, explore protective factors, theoretical models, and intervention approaches aimed at enhancing personal growth and mental health across diverse populations and settings. The following section offers a thematic synthesis of the key contributions.

## 2 Thematic overview

The 10 studies included in this Research Topic are grouped into four thematic areas. The first study explores how personal competencies promote psychological wellbeing in young people during formative stages. The second study focuses on the effects on teachers, particularly in relation to reducing burnout, supporting career decision-making, and managing stress. The third study includes contributions that highlight the influence of cultural identity, emotional intelligence, and critical thinking in populations engaged in arts, music, or sports. The fourth study presents a methodological contribution involving the validation of an assessment instrument.

A total of four studies explore the wellbeing of young adults during their higher education years. For example, Pinos Ullauri et al. analyzed how postgraduate studies influence the development of soft skills in engineering students. They found that, on average, these programs enhance soft skills, particularly leadership, innovation, and stress management. Huang et al. found that self-compassion and stress management are associated with higher life satisfaction, an effect mediated by personal competencies such as resilience, optimism, and self-efficacy. Fan and Cui demonstrated that mindfulness, self-efficacy, and self-regulation predict wellbeing among university students learning English as a foreign language, with self-regulation serving as a key mediator. Li F. et al. found that social media usage influences the career decision-making of university students through the mediation of work values and, notably, self-efficacy, which plays a critical role in enhancing this effect.

Another thematic area is represented by three studies that address teacher burnout and performance. One study, conducted by Chunyan and Ying with English teachers in primary schools, showed that a proactive personality predicts greater enjoyment in teaching and lower levels of burnout. Furthermore, Li Y. et al. explored how work–family conflict is associated with teacher burnout, with this relationship mediated by depression and moderated by cognitive reappraisal.

Finally, some studies highlight the impact of culture and identity on psychological development. Peng investigated the relationship between critical thinking, cultural intelligence, and eudaimonic wellbeing among art students, providing evidence of their role in the development of personal identity. In a study on music, Jing found that emotional intelligence mediates the effect of stress on mental health in music students, with this relationship moderated by the honesty–humility trait from the HEXACO model. In the context of sports, Liang et al. revealed that mindfulness helps reduce impulsivity in athletes by enhancing self-reflection and coping skills.

From a methodological perspective, the contribution by Doshi et al. validates the Swedish version of the Impostor Profile (IPP30) scale for assessing the impostor phenomenon, opening new avenues for understanding its impact on self-perception and emotional adjustment.

## 3 Conclusion and future directions

The studies gathered in this Research Topic reinforce the idea that psychological wellbeing is built through personal competencies and cultural experiences that help individuals confront contemporary challenges, strengthen their identity, and support integral development. Personal competencies such as self-compassion, self-efficacy, critical thinking, and emotional intelligence have positive effects on both student and teacher populations, acting as protective factors against stress, impulsivity, and burnout. In addition, the role of cultural, artistic, and contextual elements in shaping wellbeing is evident, offering a broader and more sensitive perspective on the diversity of experience. This Research Topic of studies not only expands our understanding of the mechanisms underlying wellbeing and personal development but also provides practical tools for educators, psychologists, and professionals.

Looking ahead, there is a need for longitudinal studies that capture the dynamic evolution of these competencies over time. Equally important is the development of training programs and interventions that integrate these variables across educational, professional, and community settings, with the aim of not only preventing psychological distress but also promoting personal and social development. This Research Topic also highlights the importance of advancing innovative methodological approaches that can provide deeper insights into these complex processes.

